# Selective Autophagy Receptor p62/SQSTM1, a Pivotal Player in Stress and Aging

**DOI:** 10.3389/fcell.2022.793328

**Published:** 2022-02-14

**Authors:** Anita V. Kumar, Joslyn Mills, Louis R. Lapierre

**Affiliations:** Department of Molecular Biology, Cell Biology and Biochemistry, Brown University, Providence, RI, United States

**Keywords:** p62 (sequestosome 1(SQSTM1)), autophagy, proteasome, aging, neurodegenerative diseases

## Abstract

Efficient proteostasis is crucial for somatic maintenance, and its decline during aging leads to cellular dysfunction and disease. Selective autophagy is a form of autophagy mediated by receptors that target specific cargoes for degradation and is an essential process to maintain proteostasis. The protein Sequestosome 1 (p62/SQSTM1) is a classical selective autophagy receptor, but it also has roles in the ubiquitin-proteasome system, cellular metabolism, signaling, and apoptosis. p62 is best known for its role in clearing protein aggregates via aggrephagy, but it has recently emerged as a receptor for other forms of selective autophagy such as mitophagy and lipophagy. Notably, p62 has context-dependent impacts on organismal aging and turnover of p62 usually reflects active proteostasis. In this review, we highlight recent advances in understanding the role of p62 in coordinating the ubiquitin-proteasome system and autophagy. We also discuss positive and negative effects of p62 on proteostatic status and their implications on aging and neurodegeneration. Finally, we relate the link between defective p62 and diseases of aging and examine the utility of targeting this multifaceted protein to achieve proteostatic benefits.

## 1 Introduction

The health and survival of an organism is reliant on efficient proteostasis, and breakdown of this process results in accumulation of toxic protein aggregates that contribute to aging and age-related diseases (Kaushik and Cuervo, 2015). The main contributors to protein quality control include chaperones for protein folding and the ubiquitin-proteasome system (UPS) and autophagy for protein degradation. The UPS degrades individual proteins with specific polyubiquitin tags including short-lived, misfolded, and damaged proteins (Rock et al., 1994), while autophagy has the capacity to degrade large proteins as well as protein aggregates and damaged organelles (reviewed by Lamb et al., 2013). Autophagy is enhanced as a compensatory mechanism for impaired proteasomes and coordination between the UPS and autophagy ensures efficient protein turnover (reviewed by Dikic, 2017). Sequestosome 1 (SQSTM1 or p62), hereafter p62, a ubiquitous and multifunctional protein, can direct ubiquitinated proteins to the proteasome (Babu et al., 2005; Myeku and Figueiredo-Pereira, 2011) or the growing autophagosome (Pankiv et al., 2007), highlighting its role as a key receptor and pivot for the two main cellular pathways of protein degradation. Here, this review discusses new knowledge in p62 biology with a focus on the role of p62 during cellular stress and aging, and in age-related diseases.

## 2 Roles of p62

**FIGURE 1:**
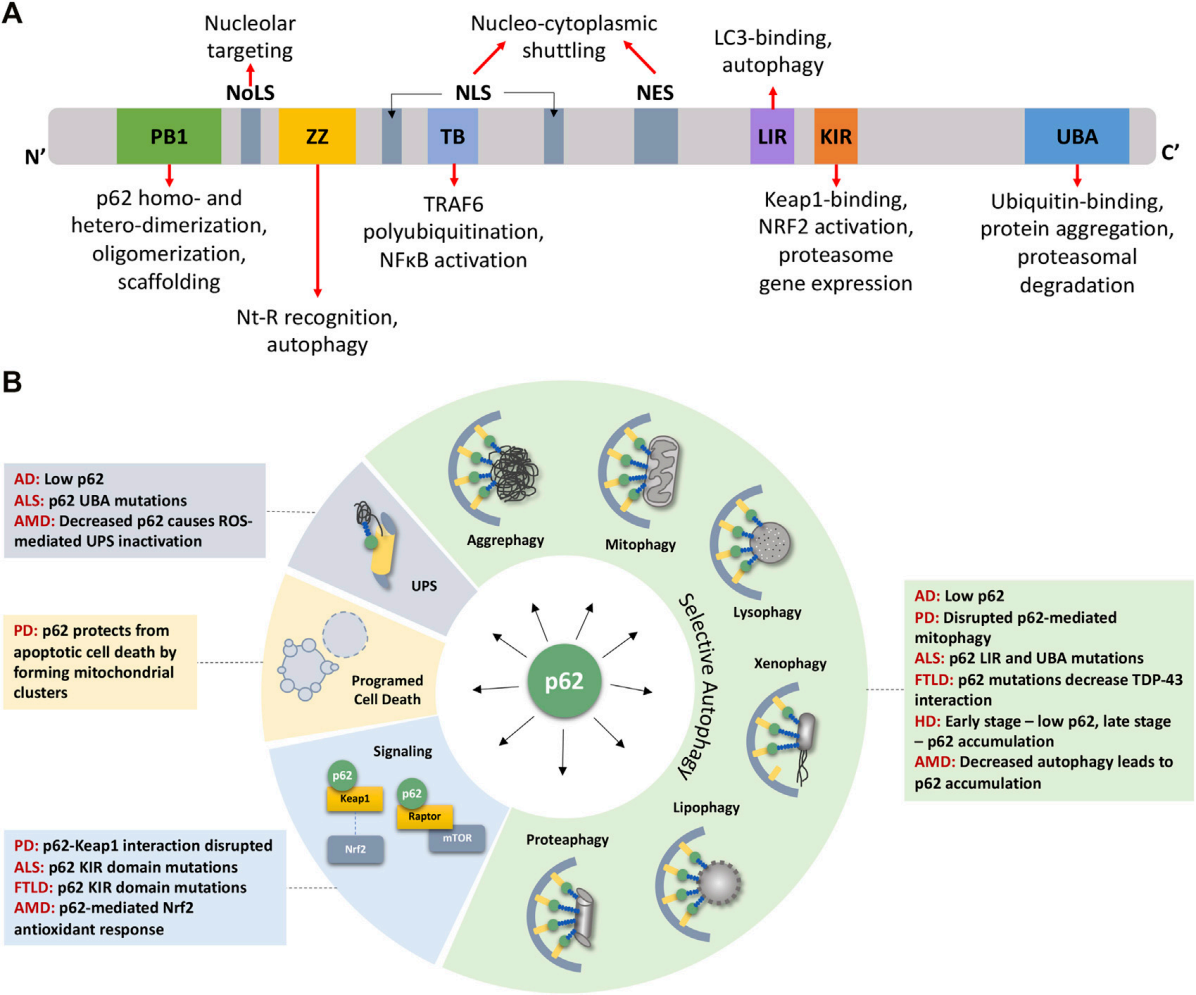
p62 domains, its multifaceted nature, and its impact on detriments associated with age-related degenerative diseases. **(A)** p62 protein consists of several well-characterized domains that interact with various proteins leading to p62’s involvement in diverse functions (see text for details). **(B)** p62 plays roles in various forms of selective autophagy, the UPS, programmed cell death, and signaling pathways. These functions are disrupted owing to mutations or aberrant expression/accumulation of p62 in several age-related degenerative diseases discussed in this review. AD Alzheimer’s Disease, ALS Amyotrophic Lateral Sclerosis, AMD Age-related Macular Degeneration, FTLD Frontotemporal Lobar Degeneration, HD Huntington’s Disease, NES Nuclear Export Sequence, NLS Nuclear Localization Sequence, NoLS Nucleolar Localization Sequence, PD Parkinson’s Disease.

p62 has multiple conserved domains that interact with various proteins with diverse functions (Figure 1A). These domains and associated post-translational modifications have been discussed in detail in several excellent reviews (Lin et al., 2013; Emanuele et al., 2020; Berkamp et al., 2021). From N- to C-terminal, these domains include the PB1 domain for p62 homo- and hetero-dimerization and oligomerization (Moscat et al., 2006; Nakamura et al., 2010; Christian et al., 2014; Ciuffa et al., 2015; Turco et al., 2021), the ZZ domain that recognizes N′-end degrons in autophagic substrates (Cha-Molstad et al., 2017; Kwon et al., 2018), a TRAF6 binding (TB) domain (Wooten et al., 2005), the LC3- and Keap1-interacting regions (LIR and KIR, respectively) (Pankiv et al., 2007; Ichimura et al., 2013), and the ubiquitin-binding UBA domain (Isogai et al., 2011). Flanking the TB domain lie nuclear localization and nuclear export signal (NLS and NES) sequences which mediate the nucleo-cytoplasmic shuttling of p62. While p62 aggregates with cytoplasmic inclusions containing ubiquitinated proteins, nuclear p62 associates with nuclear polyubiquitinated proteins at promyelocytic leukemia (PML) bodies and accumulates when nuclear export mediated by the exportin XPO1 (CRM1) is blocked (Pankiv et al., 2010). Nuclear p62 can form condensates with ubiquitinated proteins to degrade nuclear proteins *via* the nuclear UPS machinery (Fu et al., 2021). A nucleolar localization sequence (NoLS) has recently been identified between the PB1 and NLS regions that causes p62 to shuttle to the nucleolus where it sequesters nuclear proteins during cellular stress (Lobb et al., 2021). Overall, p62’s domains provide a scaffold that directs substrates to autophagosomes and facilitates the autophagic process. For instance, formation of helical p62 filaments by polymeric PB1 self-assembly facilitates autophagic cargo uptake (Jakobi et al., 2020). The ZZ domain that recognizes N-degrons such as N-terminal arginine (Nt-R) mediates p62 puncta formation and autophagy (Zhang et al., 2018). The UBA domain has important phosphorylation sites S403 and S409 that, when phosphorylated, increase p62’s affinity for polyubiquitin chains (Matsumoto et al., 2011; Lim et al., 2015). In addition to autophagy, some domains cause p62 to participate in signaling events, for e.g., p62’s TB domain triggers TRAF6 polyubiquitination thereby activating the inflammatory NF-κB pathway (Wooten et al., 2005; Zotti et al., 2014). The KIR domain interacts with Keap1 which releases the transcription factor Nrf2 to translocate to the nucleus and stimulate expression of p62 and antioxidant element-responsive and proteasomal genes (Ichimura et al., 2013; Sha et al., 2018). Altogether, p62 levels and its multiple interactions have important ramifications in the onset of aging where UPS and autophagic capacities progressively decline.

### 2.1 p62 and Selective Autophagy

p62 is the first selective autophagy receptor to be characterized (Bjorkoy et al., 2005; Pankiv et al., 2007). Its transcription is modulated by the conserved autophagy and lysosomal regulator transcription factor EB (TFEB) (Sardiello et al., 2009; Settembre et al., 2011; Lapierre et al., 2013), whose nuclear localization is modulated by major nutrient sensor mTORC1 (Pena-Llopis et al., 2011; Martina et al., 2012) and nuclear export protein XPO1 (Kirli et al., 2015; Silvestrini et al., 2018). The oxidative stress transcription factor Nrf2 also induces p62 expression (Jain et al., 2010). One of the key roles of p62 is to deliver various ubiquitinated cargoes bound to its UBA domain to the autophagosome *via* LIR domains, ultimately leading to their degradation by the lysosome (Bjorkoy et al., 2005; Liu et al., 2016). Defective autophagy leads to p62 accumulation, and p62 levels are used as a marker for autophagic flux, along with LC3B (Mizushima et al., 2010). During recognition of aggregated polyubiquitinated cargo, p62 self-assembles and forms oligomers, resulting in clearance of misfolded proteins by a process known as aggrephagy (Wurzer et al., 2015; Galluzzi et al., 2017; Zaffagnini et al., 2018). p62 forms cytosolic inclusion bodies known as p62 bodies consisting primarily of K63-linked polyubiquitinated substrates (Bjorkoy et al., 2005; Stolz et al., 2014). Polyubiquitinated cargoes linked *via* lysine-63 (K63) are more apparent in p62 cluster formation than K48-linked cargoes, suggesting the K63-linked chains specifically triggered clustering, while K48-linked chains needed higher concentrations for clustering, which might occur during proteasomal inhibition (Zaffagnini et al., 2018). Recently, interaction of the chaperone UTX with a Lim-binding domain of p62 was found to increase clustering and p62 body formation (Yoon et al., 2021). Notably, p62 bodies have liquid-like properties formed by polyubiquitin chain-triggered phase separation (Sun et al., 2018; Zaffagnini et al., 2018). While originally believed to be rigid, p62-ubiquitinated protein clusters are dynamic structures in which ubiquitinated proteins can freely move within them (Sun et al., 2018). The formation of p62 bodies is mediated by autophagy receptor NBR1, which activates Nrf2 and promotes Nrf2-mediated stress response (Yang et al., 2019; Sanchez-Martin et al., 2020). The oligomerization of p62 during aggrephagy, and its consequent binding to LC3B and GABARAP, is negatively regulated by the binding of short, non-coding RNA called vault RNA1-1 to p62 (Horos et al., 2019). Thus, understanding p62 cluster dynamics and regulation could further shed light onto the range of effects of p62 in aging and diseases.

In addition to its classical role as an aggrephagy receptor, p62 is involved in several other forms of selective autophagy. Mitochondrial proteins that are damaged beyond the capacity of the unfolded protein response and mitochondria losing membrane potential (Chen et al., 2020) can be autophagically degraded via mitophagy (Geisler et al., 2010). p62 is recruited to ubiquitinated outer mitochondrial membrane proteins in Parkin-dependent mitophagy and has a role in mitochondrial ubiquitination in PARKIN-independent mitophagy (Narendra et al., 2010; Yamada et al., 2018; Yamada et al., 2019). Lipid droplets, in addition to being lipid storage organelles, are emerging as hubs of cellular proteostasis integrating cytosolic and ER-related degradation processes (Roberts and Olzmann, 2020). The selective engulfment of lipid droplets (LDs) is mediated by the autophagic machinery in a process called lipophagy (Singh et al., 2009; Roberts and Olzmann, 2020). p62-mediated autophagy targets LDs for autophagic turnover in myocytes (Lam et al., 2016), hepatic cells (Wang et al., 2017; Yan et al., 2019), and macrophage foam cells (Robichaud et al., 2021), highlighting p62 as a potential receptor for LD turnover. Xenophagy, or the targeted clearance of foreign entities such as invading pathogens by autophagy, is an important part of host immune defense (Sharma et al., 2018). Phosphorylated p62 promotes ubiquitin conjugation to xenophagy target proteins (Tsuchiya et al., 2018) and *Mycobacterium tuberculosis* protein (Chai et al., 2019). Targeted autophagic degradation of the proteasome itself, termed proteaphagy, occurs in mammalian cells in response to amino acid starvation (Marshall et al., 2016; Cohen-Kaplan et al., 2017). Ubiquitinated proteasomes are recognized and recruited for autophagosomal uptake by p62 *via* its UBA domain independent of its PB1 domain (Cohen-Kaplan et al., 2017) or could be partially sequestered into aggresomes (Choi et al., 2020). Damaged lysosomes and harmful products of lysosomal rupture are cleared by lysophagy (Yim and Mizushima, 2020), and p62 is the major receptor discovered to play a role in lysophagy. It is present on damaged lysosomes along with ubiquitin-targeted AAA + -ATPase p97, and p62 deletion impairs lysosome clearance (Papadopoulos et al., 2017). p62 is also a receptor for selective autophagy of peroxisomes, termed pexophagy, where p62 interacts with NBR1 to promote clustering of peroxisomes and enhance pexophagy (Deosaran et al., 2013; Germain and Kim, 2020). Owing to its involvement in selective autophagy of several organelles, perturbance of p62 could result in accumulation of different damaged organelles commonly observed in stress, aging, and age-related diseases.

### 2.2 p62 and the Ubiquitin-Proteasome System

The Ubiquitin-Proteasome System (UPS) accounts for nearly 80% of protein degradation in the cell (Lee and Goldberg, 1998). p62 colocalizes with proteasomes (Seibenhener et al., 2004; Myeku and Figueiredo-Pereira, 2011) and can shuttle polyubiquitinated substrates for degradation via the proteasome (Babu et al., 2005). While the UBA domain on p62 recognizes ubiquitinated substrates, the PB1 “oligomerization” domain interacts with Rpn10 and Rpn1, proteins of the regulatory 19S subunit of the proteasome, to facilitate delivery of ubiquitinated substrates to the proteasome by p62 (Seibenhener et al., 2004). Additionally, with the help of its two NLS domains, p62 enters the nucleus, where it has been shown to phase separate into p62 foci which recruit functional proteasomes that actively degrade nuclear proteins and unincorporated proteasome subunits (Pankiv et al., 2010; Fu et al., 2021). Formation of such condensates is responsive to various stressors (Fu et al., 2021) and could be important in recruiting components of the UPS machinery thus improving efficiency of degradation. Inhibition of proteasome activity stimulates p62 transcription along with that of proteasomal genes (Sha et al., 2018). p62 overexpression in presence of autophagy inhibition hampers proteostatic flux through the UPS without affecting proteasome catalytic activity (Korolchuk et al., 2009) indicating a block in delivery of ubiquitinated substrates to the proteasome by p62.

### 2.3 Bridging the Ubiquitin-Proteasome System and Autophagy

The UPS and autophagy are two major intracellular degradation routes and p62 is a key mediator of crosstalk between these pathways (Liu et al., 2016). Proteasome inhibition leads to proteotoxic stress that promotes p62 phosphorylation at S403 in humans. This stabilizes ubiquitinated proteins in p62 clusters and promotes their clearance by autophagy (Matsumoto et al., 2011; Lim et al., 2015). Reducing proteasomal capacity by knocking down UPS ubiquitin receptors, PSMD4 and ADRM1, stimulates the transcription of p62 via the transcription factor ATF4 and induces compensatory autophagy (Demishtein et al., 2017). Similarly, p62 can also be induced by transcription factor Nrf1 upon pharmacological inhibition of the proteasome, which promotes cell survival by sequestering ubiquitinated proteins into inclusions (Sha et al., 2018). Upregulation of the deubiquitinase TRIM44, that binds K48-linked ubiquitin chains, promotes p62 oligomerization (Lyu et al., 2021). Prolonged proteasomal inhibition and ubiquitin overexpression causes accumulation of ubiquitinated p62 that activates autophagy (Peng et al., 2017).

Like the UPS, inhibiting autophagy also causes accumulation of p62, but this accumulation delays delivery of ubiquitinated proteins to the proteasome and thus reduces flux through the UPS (Korolchuk et al., 2009). Autophagy inhibition can also impair proteasomal function by affecting proteaphagy, in which p62 recognizes ubiquitinated proteasomes, especially prevalent during starvation, and targets them for autophagic degradation (Marshall et al., 2016; Cohen-Kaplan et al., 2017). Although p62 primarily carries out aggregation-dependent clearance of damaged material, p62 can turn detrimental by exacerbating pathological aggregation and proteotoxicity during autophagy inhibition or when proteostasis is overwhelmed. Since p62 is an important receptor that delivers substrates for both proteasomal degradation and autophagy, alterations in p62 levels and function could influence the activity of UPS versus autophagy.

### 2.4 Additional Roles of p62

In addition to its well-studied role in autophagic and proteasomal degradation, p62 influences other cellular pathways, including pathogen resistance, programmed cell death, and signal transduction, through its scaffolding property brought about by its several interacting domains (Figure 1A). Along with other autophagy components such as LC3, ATG7, and ATG16L1, p62 restricts the growth of *Toxoplasma gondii* by encapsulating them in vesicles that do not fuse with lysosomes (Selleck et al., 2015). In addition to pathogen resistance, p62 also controls programmed cell death independent of autophagic cargo degradation. By recruiting RIPK1, a component of the necroptosis complex, p62 controls a switch from apoptosis to necroptosis in a prostate cancer model (Goodall et al., 2016). Due to its ability to provide scaffolding, p62 participates in several signal transduction cascades by bringing together pathway components (Bitto et al., 2014). Briefly, it facilitates TNF-R and IL-1βR signaling, activating the NF-κB pathway (Sanz et al., 1999; Wooten et al., 2005), enables the oxidative stress response by binding to Keap1 which allows the release of Nrf2 to induce the antioxidant response (Ichimura et al., 2013), and stimulates apoptosis by acting downstream *via* cullin-3 regulation of caspase-8 (Jin et al., 2009). p62 also participates in amino acid sensing by the mTORC1 pathway, which is perhaps most relevant to stress and aging. p62 associates with components of the mTORC1 complex, Raptor and Rag GTPases, which sense amino acid levels and activate mTORC1 (Duran et al., 2011). Since mTORC1 signaling regulates autophagy, p62 can influence the balance between autophagy and cell growth by its action on finetuning mTORC1 signaling (Moscat and Diaz-Meco, 2011; Komatsu et al., 2012). Through its involvement in signaling pathways governing cell growth and autophagy, p62 is an important player in tumor initiation and progression (Moscat et al., 2016; Hennig et al., 2021). Altogether, owing to its multifaceted nature and ability to modulate growth and survival mechanisms, p62 plays a pivotal role in cellular stress, aging, and various pathologies including metabolic and neurodegenerative diseases (Figure 1B).

## 3 Contribution of p62 Dynamics to Aging

p62 contributes to many cellular processes, so in theory, mutations, loss, or mislocalization of p62 is bound to trigger various outcomes, and the context will determine its impact on health (positive or negative). p62 expression is age- and disease-dependent, characterized by a decline of expression with age and senescence in mice (Kwon et al., 2012; Salazar et al., 2020) and flies (Aparicio et al., 2019), as well as a decrease in human and mouse brains with Alzheimer’s disease (Du et al., 2009). To this end, few p62 mutations that lead to disease have been identified, such as those in Paget’s disease of bone and Amyotrophic Lateral Sclerosis (ALS) (Kwok et al., 2014; Seton et al., 2016; Ma et al., 2019); however, the effects due to complete loss, overexpression, protein-protein interaction disruptions, or mislocalization of the protein have been explored experimentally. Much of the research investigating p62’s role has been done in p62 null or p62 overexpression backgrounds in different species, with only a few studies investigating the tissue-specific or spatio- and temporal-specific roles of the p62 protein. This section will cover what is known about the requirement of p62 for lifespan and disease prevention, how the timing, location, and protein levels are essential to the overall health of the organism, and the role of p62 in specific age-related diseases (Figure 1B).

### 3.1 Positive Role of p62 in Lifespan and Healthspan

A number of studies have shown a beneficial lifespan response to p62 overexpression, albeit with context-specific caveats. For instance, p62 overexpression has been shown to prolong lifespan in *Drosophila melanogaster*, but only in females that have the overexpression initiated at middle-age. This follows the endogenous expression pattern: transcript levels of p62 are increased in early adulthood, but the sharp decrease in expression after midlife can be rescued by p62 overexpression only at that stage (Aparicio et al., 2019). There is no effect on lifespan when p62 is overexpressed in early adulthood, and it is not clear why there is a sex-dependent benefit. In another example, *Caenorhabditis elegans* shows an extension of lifespan with the overexpression of SQST-1/p62, similar to the lifespan extension seen with hormetic heat shock. However, this lifespan extension is impaired by the loss of neuronal *sqst-1/p62*. Further, only the nerve-ring neurons require SQST-1/p62 for autophagosome formation (Kumsta et al., 2019). This study indicates a potential tissue-specific requirement of SQST-1/p62, which reveals differential benefits or detriments depending on the tissue target.

Other studies have demonstrated the importance of p62 by investigating the effects of p62 knockout. For example, loss of p62 in the pituitary has a detrimental effect on female mouse fertility due to impaired luteinizing hormone production through mitochondrial OXPHOS signaling (Li X. et al., 2021). Another study showed that p62 protects against glycation-derived toxicity by driving the autophagic degradation of harmful age-associated advanced glycation end products (Aragones et al., 2020). Also, loss of p62 increases the rate of aging by inducing senescence through downregulation of autophagy in vascular smooth muscle cells, suggesting a protective role of p62 in vascular disease and atherosclerosis (Salazar et al., 2020). Many other studies have found that p62 maintains health by mediating the oxidative stress response through interactions with other proteins. Notably, p62 interacts with Keap1 (Figure 1A), which prevents its inhibitory binding to Nrf2. Increased p62 leads to hyperactivation of Nrf2 target genes, which protect against oxidative damage and inflammation. The interaction between p62 and Keap1 declines with age and is lost in some neurodegenerative diseases, leading to age-associated oxidative damage and inflammation (Ma et al., 2019). Further adding to this decline is the oxidative damage to the p62 promotor, demonstrated in cells treated with H_2_O_2_, which yields lower p62 levels (Du et al., 2009). Another recent finding shows that treatment with spermidine, a lifespan-extending polyamine, upregulates p62 expression, and this induces cytoprotective autophagy of female germline stem cells (FGSCs) *ex vivo*. The upregulation of p62 by spermidine is indispensable to delay aging caused by oxidative stress-induced senescence (Yuan et al., 2021). Interestingly, p62 bodies can form “gel-like” droplets, which serves as a platform for the anti-oxidative stress response by sequestering Keap1 within the droplets (Kageyama et al., 2021). Overall, many studies support that the loss of p62 is generally detrimental due to its protective role in the oxidative stress response.

### 3.2 Negative Role of p62 in Lifespan and Healthspan

While it is evident that p62 plays an important role in cellular homeostasis through proteostasis and signaling pathways, it is important to highlight that elevated levels of p62 can also be detrimental. A number of studies demonstrate the harmful effects of p62 accumulation through loss of autophagy, such as the loss of oxidative stress response *via* FOXO1/3 (Zhao et al., 2019). Other studies show that overexpression or general induction of p62 can be damaging. For instance, tumorigenesis is associated with increased p62 (Mathew et al., 2009), or inflammation is triggered by the interaction between p62 and αPKCs to activate the NF-kB pathway which induces senescence (Ma et al., 2019). Genetic overexpression of p62 can have unfavorable effects as well. Indeed, the UPS pathway of degradation does not respond to p62 overexpression efficiently; it is not sufficient to increase proteasome activity, but it instead delays UPS substrate delivery, causing proteotoxic stress that eventually activates the autophagic pathway (Korolchuk et al., 2009). Finally, our group has found that lifelong SQST-1/p62 overexpression in *C. elegans* maintained under mild heat stress (25°C) causes SQST-1/p62 to accumulate, leading to a decrease in lifespan (Kumar et al., 2021). This shortened lifespan in the SQST-1/p62 overexpressing strains is due to the marked upregulation in SQST-1/p62 transcription and protein levels which exacerbates the proteotoxic stress of accumulated ubiquitinated proteins. Notably, lipid droplet accumulation restores SQST-1/p62 function and dynamics and prevents the rapid proteostatic decline. Overall, these studies highlight the need to reassess the idea that enhancing the expression of a single autophagy receptor, such as p62, is necessarily beneficial for proteostasis and lifespan, especially when the whole process of autophagy, involving more than 30 proteins (Wong et al., 2020a), is not enhanced concomitantly.

## 4 The Role of p62 in Age-Related Diseases

### 4.1 Neurodegenerative Diseases

#### 4.1.1 Alzheimer’s Disease

Alzheimer’s Disease (AD) is a progressive neurodegenerative disease that destroys memory and cognitive functions and is characterized by accumulation of amyloid-β (Aβ) and hyperphosphorylated tau, causing amyloid plaques and tau tangles, respectively, in the brain. Low expression of p62 has been observed in the frontal cortex of AD patients as well as in transgenic AD mouse models; however, the remaining p62 is associated with tangles, and is believed to play an important role in tau degradation (Salminen et al., 2012). In addition to decreased tau clearance, the low levels of p62 also lead to decreased Nrf2-dependent antioxidant response (Ma et al., 2019), suggesting that impaired oxidative stress resistance may significantly contribute to AD pathology.

#### 4.1.2 Parkinson’s Disease

Parkinson’s Disease (PD) is characterized by intracellular accumulation of Lewy bodies and Lewy neurites, which consist of p62-associated aggregated proteins, including α-synuclein, parkin, and ubiquitinated proteins (Shin et al., 2020). Disrupted p62-mediated mitophagy due to mutations in PINK and parkin is the most common cause of familial PD (Geisler et al., 2010). Mutations in the kinase LRRK2 disrupt the p62-LRRK2 interaction and impairs LRRK2-mediated phosphorylation of p62 and LRRK2 degradation. Phosphorylated p62 cannot interact with Keap1, which allows Keap1 to inhibit Nrf2 signaling, connecting oxidative stress to PD pathology (Park et al., 2016). Hyperactivation of Parkin/PINK1 mitophagy is also implicated in PD pathogenesis, but recent research suggests that p62 could prevent apoptotic cell death by clustering mitochondria to regulate this process (Xiao et al., 2017).

#### 4.1.3 Amyotrophic Lateral Sclerosis

Amyotrophic Lateral Sclerosis (ALS) is characterized by ubiquitin-p62 positive intraneuronal inclusions, with increased levels of p62 in the spinal cord and motor neurons. Some cases of ALS are associated with p62 mutations in the UBA, LIR, or KIR domains. UBA domain mutations prevent interactions with ubiquitinated proteins tagged for degradation (Ma et al., 2019), and LIR domain mutations lead to reduced LC3 binding, both causing decreased p62-mediated cargo degradation (Deng et al., 2020). Finally, KIR domain mutations disrupt the interaction with Keap1, deactivating Nrf2 and preventing an effective response to oxidative stress, which may contribute to the etiology of ALS (Goode et al., 2016). There is also evidence that p62 itself might intensify ALS. For instance, autophagic induction by rapamycin treatment exacerbated the pathology in an SOD mutant mouse model of ALS, probably due to apoptosis and oxidative stress (Zhang et al., 2011). Additionally, a *C. elegans* model of ALS showed defective autophagy and increased levels of p62; however the removal of p62 alleviated the locomotion defect without restoring the autophagy defects (Baskoylu et al., 2022), suggesting that the autophagy defects are upstream and not dependent on p62 in *C. elegans*.

#### 4.1.4 Frontotemporal Lobar Degeneration

Frontotemporal Lobar Degeneration (FTLD) is characterized by neuronal cytoplasmic inclusions of TDP-43, which are correlated with neurodegeneration. Overexpression of p62 leads to the mislocalization of TDP-43 to the cytoplasm, causing aggregates and neuronal death (Foster et al., 2021). p62 has also been found in TDP-43-negative inclusions in a subset of FTLD patients (Al-Sarraj et al., 2011), opening the possibility that other protein aggregates contribute to FTLD pathogenesis. p62’s interaction with these aggregates suggests their degradation is mediated by p62, and mutations in p62 that decrease this interaction or any disruption in the autophagic or UPS pathways could possibly lead to the accumulation of these inclusions. Notably, the same KIR mutation in ALS is also seen in FTLD cases (Ma et al., 2019).

#### 4.1.5 Huntington’s Disease

Huntington’s Disease (HD) is characterized by neuronal degeneration associated with a CAG repeat expansion (polyQ) in the huntingtin gene (mHTT). Early work investigating mTOR inhibition by rapamycin treatment in a HD mouse model demonstrated reduce HD pathology (Ravikumar et al., 2004); however, later work shows that it is independent of autophagy and is instead due to decreased protein synthesis (King et al., 2008). In fact, autophagy is already upregulated in HD, but the defect lies in the recognition of autophagic cargo, not the process itself (Martinez-Vicente et al., 2010). Therefore, the inability for autophagosomes to recognize cargo for degradation (Martinez-Vicente et al., 2010) and p62’s sequestration of ULK into an insoluble cellular fraction (Wold et al., 2016) are likely responsible for the dysfunctional autophagy seen in HD cells. The role of p62 was investigated in a Huntington mouse model which revealed that levels of p62 were reduced in all brain regions at early stages of the disease, but then accumulated in striatal and hippocampal neurons in the late stage of the disease, in particular in the nuclei of these cells. Indeed, the increased p62 accumulated with mHTT in neuronal nuclei (Rue et al., 2013). Thus, p62 depletion reduces nuclear inclusions and ameliorates HD (Kurosawa et al., 2015), but reduction of p62 protein levels or dysfunction of p62 significantly increased cell death induced by mHTT in HD (Bjorkoy et al., 2005). Generally, p62 is upregulated in response to proteotoxic stress (Lim et al., 2015), and the subcellular localization of p62 in response to this stress may underlie the vulnerability of HD cells to cell death under proteotoxic stress (Huang et al., 2018).

### 4.2 Age-Related Macular Degeneration

Age-related macular degeneration is a progressive and degenerative eye disease, with aging and oxidative stress contributing to its pathogenesis. This disease is mainly caused by breakdown of proteostasis in Retinal Pigment Epithelium (RPE), leading to the accumulation of cellular waste such as lipofuscin. Oxidative stress induced by H_2_O_2_ leads to inhibition of proteasome activity and an increase in p62 expression in RPE cells. Silencing of p62 increases the accumulation of protein aggregates in RPE cells that showed decreased autophagy and Nrf2-mediated antioxidant response (Blasiak et al., 2019). RPE protection against oxidant-induced protein damage seems to rely on p62; however, if p62 is unable to be cleared by autophagy, the aggregates that form will likely contribute to further damage to the RPE cells, highlighting the importance of properly regulating p62 expression.

### 4.3 Cancer

p62 is commonly upregulated in human tumors (Zatloukal et al., 2007), and the multiple roles p62 plays in cancer has been recently reviewed (Hennig et al., 2021). Suppression of tumorigenesis by autophagy is accomplished by limiting p62 accumulation and preventing activation of NF-κB. p62-induced expression of NF-κB in autophagy-defective cells is sufficient to activate the DNA damage response and enhance tumor growth (Mathew et al., 2009). Further, p62 overexpression promotes bone metastasis by stimulating migration, but not proliferation, of lung adenocarcinoma. High expression of p62 was associated with poor prognosis in patients with bone metastasis (Li D. et al., 2021). p62-induced tumorigenesis reveals an important concern when considering p62 induction as a potential therapeutic *via* autophagy induction.

## 5 Conclusion

Most degenerative diseases are characterized by protein aggregation, so naturally it would seem that targeting autophagy would be the best option to clear large aggregates. But what is the best way to induce this process when it requires coordination of multiple steps for autophagy to work? Since p62 has a multitude of roles beyond protein turnover and is also not essential for autophagy (Fan et al., 2010; Xu et al., 2015), specifically targeting p62 is not necessarily the answer. Induction of the entire autophagic pathway may instead be required. Therefore, identifying the limitations of pharmacological or genetic induction of autophagy (for example, by rapamycin treatment or TFEB activation), may offer better opportunities to therapeutically harness this process.

Loss of p62 leads to accelerated aging due to a decline in proteostasis, dysregulation of signaling pathways, and inability to sufficiently respond to oxidative stress. However, overexpression of p62 has potential detrimental effects, including accumulation of cellular p62-aggregates (Kumar et al., 2021), tumorigenesis and metastasis induction (Zatloukal et al., 2007; Mathew et al., 2009; Li D. et al., 2021), and mislocalization of the protein (Kurosawa et al., 2015). In order for p62 overexpression to be beneficial, global coordination of the induction of the 30 + autophagy genes (Wong et al., 2020b) by, for example, transcription factors FOXO and TFEB (Lapierre et al., 2015) may enhance autophagy protein levels and increase autophagic flux. In addition, the upstream ubiquitination machinery, including the E3 ligases correctly linking the poly-ubiquitin (K-63), must be functional to label appropriate cargo for degradation (Ma et al., 2019). Research remains to be done to elucidate the p62-associated changes that contribute to aging (Table 1). Additionally, it will be essential to classify what effects tissue- and spatiotemporal-specific p62 expression are accountable for. Understanding how to harness the positive and avoid the negative effects of p62 is crucial before it can be seriously considered as a therapeutic target for proteinopathies and neurodegenerative diseases.

**TABLE 1:** Exploratory avenues of p62-associated changes.

Cellular process	Potential p62-associated effects	Refs
Post translational modifications		
Phosphorylation, Ubiquitination, Acetylation	p62 is under or over post translationally modified	McEwan and Dikic (2011)
	Autophagy induction, regulation, and fine tuning: p62 accumulation if autophagy dysregulated	
	Selective autophagy dysregulated		
Ubiquitination	Cargo is not appropriately ubiquitinated, then p62 will not recognize it for degradation
Phosphorylation	p62 pS407: release UBA domain	Kageyama et al. (2021)
	p62 P-S403: enhance Ub binding	
	p62 P-S349 enhances Keap1 interaction (Nrf2 activation) and FIP200 interaction	
Protein-Protein interactions		
Regulated degradation of signaling pathway components	Loss of p62 interactions with proteins that should be degraded dysregulates pathways	
	Dishevelled protein not degraded will increase Wnt signaling	Wei (2012)
	cyclic AMP phosphodiesterase-4A4 (PDE4A4) not degraded augments cAMP signaling	Ma et al. (2019)
Autophagic cargo recognition	Cargo tagged for autophagic clearance is not recognized, p62 accumulates	Martinez-Vincente et al. (2010)
Signaling pathways		
mTORC1	Overexpression of p62 causes hyperactivation of mTORC1, leading to tumorigenesis	Duran et al. (2011)
Cellular localization		
Nucleus/cytoplasmic partitioning	p62 mislocalized from the nucleus leads to decreased nuclear UPS/proteostasis	Fu et al. (2021)
Trafficking of aggregates	HDAC6 needs tubulin for trafficking, p62 accumulates if aggregates are not delivered to AP formation site	McEwan and Dikic (2011)	
Trafficking of autophagosomes (AP)	p62 accumulates in APs if not delivered to LY
Trafficking of lysosomes (LY)	p62 accumulates in APs if LY cannot localize and fuse with AP
